# Swedish Tennis Coaches’ Everyday Practices for Creating Athlete Development Environments

**DOI:** 10.3390/ijerph17124580

**Published:** 2020-06-25

**Authors:** Göran Gerdin, Per Göran Fahlström, Mats Glemne, Susanne Linnér

**Affiliations:** Department of Sport Science, Linnaeus University, 351 95 Växjö, Sweden; pergoran.fahlstrom@lnu.se (P.G.F.); mats.glemne@lnu.se (M.G.); susanne.linner@lnu.se (S.L.)

**Keywords:** athlete development environment, athlete development, tennis, Sweden, coaching

## Abstract

Finding and describing the optimal path to elite athletic performance has, for a long time, been a challenge for researchers. This study examined Swedish tennis coaches’ everyday practices for creating athlete development environments and the environmental factors that promote or hinder athlete development. The study was conducted in 2018–2019 and included in-depth focus groups with 13 Swedish full-time tennis coaches. Data were analyzed using thematic analysis and by drawing on models for studying athlete development environments. The results highlight how the coaches’ everyday work involves a range of administrative tasks, which ultimately means that there is little to no time left for focusing on athlete development. These results also draw attention to concerns about these professional coaches’ health, with increasing demands in their roles to manage administrative tasks in addition to the coaching and time spent on the court with their athletes. The results further reveal how the tennis clubs’ boards are increasingly interested in sound economy and high participation levels rather than focusing on performance outcomes and developing elite athletes. Finally, the results from this study emphasize the importance of increased collaboration and communication between clubs, coaches, regions, and the national association to create common and clearer guidelines for long-term athlete development. Future studies could engage in longitudinal and ethnographic work with tennis clubs of varying size and geographical locations, involving different stakeholders (e.g., coaches, management, parents, players) in order to further explore the environmental factors that promote or hinder athlete development.

## 1. Introduction

Finding and describing the optimal path to elite athletic performance has, for a long time, been a challenge for researchers. For decades, various researchers have studied this area, as detailed in [1,2,3,4], typically by using the two concepts of “talent identification” and “talent development”. These studies on talent identification and development have, through their influence on both coaches and athletes, come to have a great impact on how different sporting organizations and environments design their training programs [5]. However, research has highlighted the lack of consistency between groups of stakeholders (parents, coaches, and the sport’s national governing body) regarding issues related to talent identification and development [6]. For example, a study that highlighted the talent development programs of the Swedish sport federations [7] shows that these sporting organizations attach great importance and effort to identifying talents early. This often happens despite them believing that the innate characteristics of talent are not decisive in determining long-term elite performances. In addition, they emphasize the difficulty of identifying talent early when there are no recognized criteria to start with [7].

Over the years, there have been many and varying attempts at finding a definition for talent such as by Brown [8], who described talent as a ‘‘special, natural ability’’ and a ‘‘capacity for achievement or success’’, and Howe et al. ([9], p. 399) who stated, ‘‘the likelihood of becoming exceptionally competent in certain fields depends on the presence or absence of inborn attributes variously labeled as talents or gifts’’. Further, Gagne ([10], p. 11), as part of the Differentiated Model of Giftedness and Talent (DMGT) model, described talent as ‘‘the outstanding mastery of systematically developed abilities, called competencies (knowledge and skills), in at least one field of human activity to a degree that places a person at least among the top 10% of age peers who are or have been active in that field’’. The fact that many of the qualities distinguishing top athletic performance in adults may not be apparent until late adolescence [11] further adds to the complexity of talent in sport and its identification. This is also indicated by the fact that early high performance is not strongly associated with later success [12]. In their recent critique of the validity of the idea of sporting talent, Baker et al. ([13], p. 59) therefore stated that “practitioners should not focus so intently on identifying and selecting talent. Evidence suggests that if it does exist, we do not know what it looks like, and are poor predictors of athlete potential”.

Recognizing the complexity of defining what sporting talent is, as well as its identification and development, most scientists agree that both biological or genetically constrained factors (nature) and factors related to experience and learning (nurture) are important [6,14]. However, this study’s focus is not on engaging in such debate but rather to examine the sporting context (environment) in which athlete development takes place. That is, in this study, we aim to investigate the environmental factors that promote or hinder the development of any athlete (“talented” or not) within that environment to enhance their performance within a specific sport. Although this study focuses on the sport of tennis, we believe that the findings and the theoretical model used can be applied to other sport contexts.

Fahlström et al. [15], as many other researchers, such as those in [16,17], argue that athlete development is a complex and heterogeneous process that must be understood in its context. They emphasize the environment’s importance for the athlete’s development and that training programs should be characterized by a long-term perspective, although variations can be large depending on the individual athlete and the environment. The athlete development environment in which the athlete exists is often complex and consists of, among other things, coaches, parents, teachers, schools, clubs, and peers. Based on his studies on child development, Bronfenbrenner [18,19] designed an “ecological” model that described the environment as structures that interact at different levels. The model shows how an individual is influenced by the different contexts in which it spends a lot of time, (e.g., the school, the home, and the sports group). In addition, the individual is affected by other contexts in which he or she is not actually included, such as the parents’ workplace. Bronfenbrenner also describes a more comprehensive level that consists of larger cultural patterns in society. For the mutual relationship between individual and context, he uses the concept of ecology. Strachan et al. [20] argue that Bronfenbrenner’s model has also been crucial for understanding the importance of the environment in sport contexts. This ecological model has influenced sport researchers both in Scandinavia and worldwide [21,22].

Several studies on athlete development environments further highlight the role of the coaches. This is often expressed in general terms where they, together with schools, parents, other sports leaders and mentors, form a support structure that has crucial importance for the athlete’s development. For example, Augustsson [23] and Eliasson [24] refer to the “sports triangle” youth–coach–parents, as first introduced by Byrne [25]. Other researchers emphasize the importance of the coach’s knowledge and commitment, as well as an interest in the coach’s own continuous professional development [26,27]. This is reaffirmed by Gilbert and Trudel [28] and Denison et al. [29] who believe it is important that the coaches have a positive attitude towards lifelong learning in a sport that is constantly evolving, and that they always follow a reflective approach regarding their own work. Similarly, Mallet et al. [30] state that high performing coaches are a prerequisite for high performing athletes.

Studies have also shown that coaches play a significant role, not only in terms of knowledge and expertise regarding sport-specific techniques and skills, but also as a support in learning psychological abilities, such as mental skills, self-control, responsibility, and problem-solving [31]. Brown [8] points out that the development of elite performance athletes requires different types of coaches during different periods of their development and career. In the beginning, most can be coached by “local” coaches who can offer a lot of time and encouragement. After a while, however, a different coach may be needed who can help the athletes refine and improve their technique and skills [8]. Finally, according to Brown, it seems to be most successful to work with coaches who have themselves been at the international level within the specific sport. In addition, there is also research that shows the coach and other leaders are less important in phases where everything is going well and when the athlete moves on without obstacles and resistance [32]. Brown [8] believes there should not be an “overbelief” in the role of the coach. In agreement with Ericsson et al. [33], he emphasizes an athlete does not necessarily reach the top level simply by working with a skilled and experienced coach.

**Figure 1:**
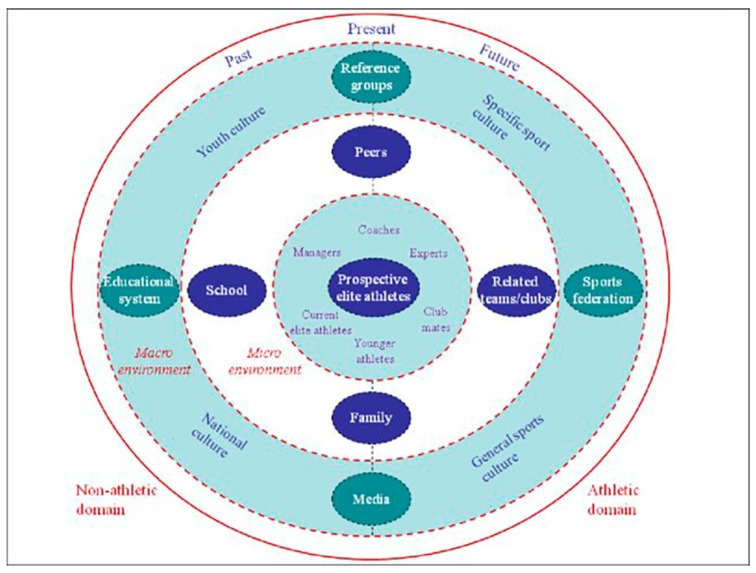
The “ATDE” model ([35], p. 39).

Alfermann and Stambulova [34] argue that the responsibility for preparing and developing successful competition athletes lies with the overall sports environment in which the athlete is located. Supported by Bronfenbrenner’s [18,19] ecological perspective on development, Henriksen [35] studied successful Scandinavian sport environments based on a “holistic, ecological approach”. The sport environments consisted of three individual sports: a sailing club in Denmark, a canoe club in Norway, and a track and field club in Sweden. Henriksen assessed a sport environment’s success using the following criteria: (1) their ability to help athletes take the step to elite sport; (2) their high reputation of external parties; (3) young athletes’ results in national competitions; (4) the amount of ambitious and happy athletes with a low drop-out rate. To study these sport environments, Henriksen [35] created the Athletic Talent Development Environment (ATDE) model, as shown in Figure 1. It builds on Bronfenbrenner’s ecological model and offers a framework for studying sport environments from a so-called holistic, ecological perspective. Similar to Bronfenbrenner’s [18,19] ecological model, Henriksen’s ATDE model comprises a psychological and cultural perspective.

According to Henriksen [35], the model serves as a guide for describing a specific sport environment and for clarifying the role, function, and relationships of different components in the environment within the talent development process. Applying the ATDE model to the three sport environments described above, Henriksen [35] identified those characteristics that the three sport environments had in common as success factors. Primarily, Henriksen’s [35] study showed the importance of the following factors: focusing on long-term development before early specialization and early successes; clear and coherent organizational culture; awareness of the ability of the environment to bring forth talents; a way to stick to values and guidelines, and to be careful when these values and guidelines change; a familial association atmosphere; basing the environment on collaboration, openness, and knowledge exchange.

**Figure 2:**
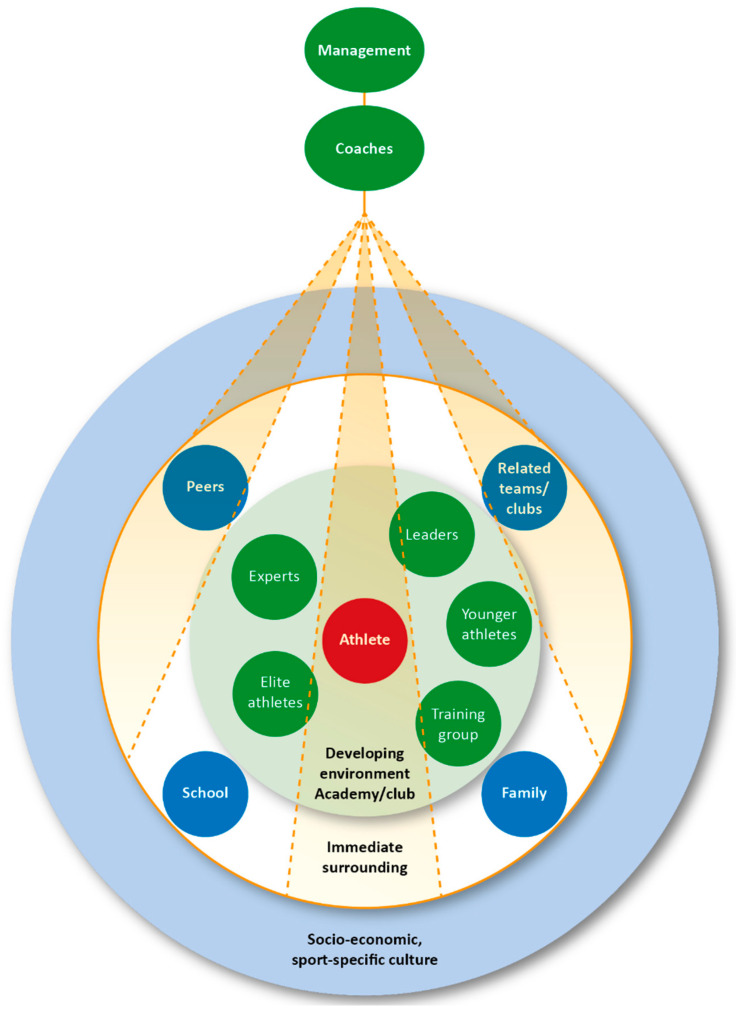
The “UPF” model ([36], p. 60).

However, Henriksen’s ATDE model is questioned by Fahlström et al. ([36], p. 21) for being somewhat difficult “to use because it contains different types of components at different levels and it is difficult to know what is more important, what affects what and what is actually influential”. With inspiration from both Bronfenbrenner and Henriksen, Fahlström et al. [36] therefore developed a refined model, which they called “Utvecklingsmiljöns påverkbara faktorer” (“the development environment’s influential factors”) also referred to as the “UPF model”, as shown in Figure 2.

The UPF model centers around the developing environment in which the athletes spend a large part of their daily life (e.g., the daily exercise/training regime). This level is characterized by communication and interaction with other people surrounding the athlete. Outside this central level there are family, friends, school, and other clubs/teams. These can interact in various ways with the athlete and his or her immediate surrounding environment. In the model there are various factors that affect the athlete rather indirectly, such as socio-economic and sport-specific culture factors (e.g., national political and socio-cultural contexts, media, and sport federations).

In order to clarify how coaches’ competencies, skills, and practices affect the culture, the climate, and the working methods in the training groups, and how facilities and resources are used etc., the coaches are placed above the other factors in the UPF model. Above the coaches, there is also a management level. Depending on the context, this level may consist of the board, club manager, academy manager, sports committee, sports manager, mentor, or any other professional or non-profit function. By placing the management level at the top of this model, Fahlström et al. [36] wanted to emphasize the importance of the management level when it comes to the impact on the development environment and the environment immediately surrounding the athlete. The management creates a framework for the coaches’ practices, recruits coaches whose knowledge and skills fit into the sporting environment, and ensures the quality of the coaches’ qualifications and professional development.

In summary, research into the development of elite athletes has recently increased to investigate the importance of the environment within this process. Indeed, based on findings in learning and development research over the past 25 years, which has demonstrated the strong positive relationship between time spent in high quality (e.g., deliberate) practice and improvements in skill [37], Baker et al. [13] even claim that the quality of the talent development environment is arguably more important to long-term success than talent identification. However, they also point at the paucity of research that examines how coaches and other stakeholders actively create these environments for exceptions, as detailed in [5,38,39].

The aim of this study is to investigate Swedish tennis coaches’ everyday practices for creating athlete development environments. More specifically, the study aims to employ Henriksen’s ATDE [35] and Fahlström et al.’s UPF [36] models to examine what environmental factors promote or hinder athlete development in Swedish tennis clubs, according to professional coaches. Accordingly, this study is guided by the following research questions: (i) What are the everyday practices of Swedish tennis coaches for creating athlete development environments? and (ii) What environmental factors promote or hinder athlete development in Swedish tennis clubs?

## 2. Materials and Methods

### 2.1. The Focus Groups

The method used in this study comprised qualitative semi-structured interviews [40] in the form of focus groups with full-time tennis coaches in Swedish clubs. In previous studies, statistics on licensed competition players and rankings, with a focus on relative-age effect [41] and quantitative surveys of the coaches’ work with athlete development [42], have been used. In this study it was therefore considered important to be able to investigate Swedish tennis coaches’ work with athlete development in a deeper and more qualitative manner. Focus groups were seen as an efficient way to gather a selection of tennis coaches in one place to conduct a joint discussion on a number of central issues related to the aims and purpose of the study. Furthermore, focus groups usually lead to “richer” conversations where the exchange and discussions between the participants are as important as the questions asked by the interviewer [40]. Such rich conversations may thus result in knowledge and insights that may not arise in more traditional individual interviews. However, it also recognized that the focus group format might have affected the coaches’ responses and, with that, this study’s overall findings due to peer pressure issues, such as agreeing with the majority’s opinion [43].

### 2.2. Participants

The participants in this study consisted of coaches from clubs in the regions Stockholm and Gothenburg. These regions were selected as it was considered as a viable way to gather coaches from a range of different clubs without coaches having to make any extended journeys or use extensive time for participation. The selection can thus be seen as a form of purposive sampling [40]. The two regional managers in these two tennis regions were then asked to select two groups of 3–4 coaches per group from clubs in varying sizes and geographical location. However, it is worth pointing out that, although some of these clubs can be considered small in these regions, they are equivalent to large clubs in the rest of the country. The inclusion of coaches and clubs from these two large city regions thus affected the overall generalizability of the findings, since the coaches’ everyday practices and environmental factors identified in this study can be seen to be more applicable to large city clubs as opposed to smaller clubs in the rural areas.

A total of 16 coaches was selected, divided into eight from each region. In addition to these coaches coming from clubs with mixed sizes, the respective regional managers considered these coaches suitable for the study as they had a close collaboration and connection with the regional association and had shown commitment and ability to develop elite athletes for many years. Some of these coaches had also participated as coaches/leaders on camps and competition trips organized by the regional association, demonstrating their interest and experience in athlete development.

Of the 16 selected coaches, 13 of them finally participated, while three had to withdraw due to illness or other commitments. The coaches were between 25 and 60 years old (M = 38.22, SD = 12.35) and their professional coaching experience varied from 5 to 40 years (M = 18.31, SD = 11.72). All the coaches worked full-time at the time of the study, but some had combined roles at different clubs/associations. The coaches’ job titles included “head coach”, “operations manager”, “youth/development coach” and “competition coach”. Most of the coaches had a solid playing background as players at a high national and some even international level. Some of the coaches had both played and worked abroad for different periods in countries such as the USA, Germany, and England. Two of the coaches were born and had grown up playing tennis in other European countries. The majority had attended all or most of the Swedish Tennis Association’s (STA) coaching qualification courses and some had also studied other sports science courses at universities in Sweden or abroad.

### 2.3. Conducting the Focus Groups

The focus groups were carried out by the lead author on four different occasions during May 2018. The lead author has extensive experience both as a tennis coach and in conducting focus groups [44]. The participants had no prior knowledge of the lead author other than him being a tennis coach and researcher interested in tennis coaching and athlete development. The composition of the groups was based on the days and times that the coaches were available where some consideration was given to getting a mix of coaches from clubs of different sizes. In one region, the focus groups were conducted in a meeting room at their own office, while the location in the other region was a conference room in a centrally located hotel.

The focus groups were conducted in the form of semi-structured interviews [40], using an interview guide. The interview guide was developed based on the results presented in the study by Fahlström et al. [37] and in dialogue with representatives from the STA. In addition to background information on the participating coaches, the interview guide revolved around questions about the clubs’ activities and the coaches’ everyday work (e.g., “What does a normal day at work look like for you?” and “What tasks take up the most amount of time in your daily work?”), as well as goals and work with developing athletes (e.g., “What goals does your club have in terms of developing elite athletes?” and “How do you work with developing elite athletes?”). However, the questions in the interview guide were mainly used as prompts to introduce topics for discussion when needed in order to allow the coaches to talk and exchange ideas with each other, so as not to overly influence responses [44].

The focus groups took between 80 and 100 min and were recorded using a digital audio recorder. The interviews were conducted in Swedish (with quotes used in this article translated into English by the lead author) and no one other than the participants and the researcher was present during the interviews. The interviewer did not take any notes during the focus groups to be able to focus on both being able to ask questions and be part of the conversation [43]. However, notes were subsequently written down as a supplement to the transcription of the audio recordings. No pilot or follow-up interviews were conducted but all the participants were subsequently contacted by email asking for clarifications and confirmations of their statements from the focus groups.

### 2.4. Data Analysis

The compilation and analysis of the collected empirical data were conducted stepwise based on the transcripts of the interviews and guided by the principles of thematic analysis [45]. That is, the focus groups’ relevant parts, based on the study’s purpose, were manually transcribed in the form of shorter and longer quotes [43]. In addition to these quotes, general comments, and reflections were also written down by the lead author, which in various ways highlighted what had been said during the interviews. Once these first steps in the processing of the material had been completed, themes were generated by the lead author from the recurring aspects named by the coaches. These were further developed and merged into key themes covering different aspects of the study’s aim and purpose. Finally, this was followed by a further analysis of these key themes by all four authors in relation to Henriksen’s ATDE model [35] and Fahlström et al.’s UPF model [36]. Data saturation, or what Saunders et al. [46] would call “a priori thematic saturation”, was achieved when no new identified themes could be identified in the data that would further exemplify the theoretical underpinnings of these two models.

### 2.5. Ethical Considerations

The study was based on the Swedish Research Council’s [47] four main requirements for conducting research: the “information”, “consent”, “confidentiality”, and “usage” requirements. All participating coaches were informed before the start of the study about its purpose and central issues where the opportunity was also given to ask questions. Furthermore, it was pointed out that participation in the study was entirely voluntary and written consent for participation was confirmed before the interviews were conducted. During the interview, it was also emphasized that all participating coaches’ names would remain anonymous and that pseudonyms would be used in any publications arising from the study. It was also pointed out to the participants that the study’s results would only be used to report back to the STA and published in scientific journals and not otherwise disseminated or misused. Since all the participants were older than 16 and able to provide informed consent, as well as there being no risk of harm to the participants, no ethical approval was needed [48]. Finally, the writing of this article was guided by the “Consolidated criteria for reporting qualitative research” (COREQ) [49].

## 3. Results

In this part of the article, the results of the conducted focus group interviews with the 13 participating coaches are presented in the form of the following five themes: (1) Club and work environment; (2) Competition versus economy; (3) Everyday practices for athlete development; (4) Goals for athlete development; (5) Collaboration and communication.

### 3.1. Club and Work Environment

The introductory questions in the interviews included questions about the club’s environment in general, such as: the number of full-time coaches; the number of members; how many juniors belonged to the club; how many of the club’s juniors were considered to be “competition juniors”, that is, active as interclub and/or tournament players. Some of these approximate figures are presented below in Table 1.

In the participating clubs, the number of full-time tennis coaches ranged from one to eight. The areas of responsibility also varied, where in some clubs (mainly the smaller ones), the coaches had a more overall responsibility, while in larger clubs, such as in coach 3′s club, there was a clear division of responsibilities:

We have six full-time coaches, four coaches for the juniors, one for competition juniors and one for adult coaching.

(Coach 3)

Already, at the question of the number of full-time coaches, the coaches raised an issue related to the quality of coaching and the athlete development environment. The coaches believed that the clubs had a hard time hiring people full-time and that the large majority of the coaches worked on a part-time basis—so-called “hourly coaches”. It was common for clubs to have 10–15 of these hourly coaches in addition to the full-time coaches.

We have three full-time employees, two part-time on 60 percent and a number of hourly coaches, about 10–15… it is difficult to have full-time coaches as the need for coaches varies a lot during the day and during the year… but the quality of coaching is definitely lowered since we cannot have more full-time coaches.

(Coach 2)

We have many part-time, I am personally opposed to having part-time coaches, but always difficult with the economy... people only focus on the economy... it’s a long day to be part-time... don’t want to complain, but you don’t survive part-time…also difficult maintaining a common approach and high quality of coaching with too many part-time coaches... it is not ok.

(Coach 8)

One of the coaches mentioned how an important function and a function that takes a lot of time for the full-time coaches was to support these hourly coaches:

We have a full-time coach both Saturday and Sunday who is responsible for supporting the hourly coaches… so that we not only have 17-year-olds who are eating sandwiches and looking at their phone.

(Coach 3)

As for the participating coaches’ own roles in the clubs and the existence of a job description, this varied somewhat between the coaches, but for most coaches, and mainly those in the slightly smaller clubs, there seemed to be no clear job description:

I have no clear job description... is often about urgent needs, what is most urgent to solve... I sit on all chairs, hard to complete anything.

(Coach 1)

There is no job description... I do it myself... Got one 23 years ago but there is no new one, it remains... I argue with the board... there is no updated work description...

(Coach 11)

On the question of what an “ordinary day at work” looks like and what tasks are part of the coaches’ everyday work, this also differed, but they shared in common that all the coaches have a wide variety of tasks:

An ordinary day... check that no graffiti on the facilities, that it looks clean and fresh... if one of the coaches are sick and needs to be replaced... the phone on and “emergency” things happening... have a shop and cafe under management of the club that I am responsible for... doing court booking and member questions... then suddenly there is someone who wants to talk about his forehand.

(Coach 1)

For some coaches the wide range of tasks that they are increasingly responsible for leads to concerns about their long-term health and well-being:

A lot of tasks that do not have to do with the tennis... sometimes I get worried about my health and well-being … I have worked seven days a week for a year... if I didn’t think it was so much fun doing this job I would have stopped long ago... but how long will I last?

(Coach 10)

Another coach stated he is “passionate about tennis”, but how this also, at times, results in some “unhealthy” days at work:

I always try to make sure we have a high quality in our training program…So if someone gets sick I usually fill in for that person rather than finding someone else…I have never taken a sick-day from work…At times this has even meant standing on the court with pneumonia or sinus infection.

(Coach 12)

### 3.2. Competition Versus Economy

The conversation in the focus groups then proceeded towards the number of juniors in the clubs and how many of these can be considered as “competition juniors”. The fact that there has been a downward trend in the number of juniors who are actively playing interclub and tournaments was a recurring theme in the coaches’ responses already at the beginning of the interviews.

We have much fewer good juniors… have noticed a big difference in the last 10 years… there are fewer and fewer players who engage in a long-term commitment to competing.

(Coach 5)

However, one of the coaches believed that this may be due to the increased focus on “internal” (club) competitions:

It has been the same trend for the last 10 years, the more successful you are in organizing internal club competitions the more people there are that are content with this and do not go on with their competition tennis. We have just over 100 juniors who in some way compete in our club but then on red, orange, green levels [softer balls and smaller courts] …” juice and cookie level” … Competition peaks around 11 years old but after that many starts dropping out.

(Coach 2)

Coach 4 had another explanation why many never started playing competitions and drop out early:

We have built up an environment in Swedish tennis where it is very difficult to get started playing competitions. It is required that you are so damn good before you start to compete. We have official competitions first when the boys and girls are 10 years old. In other sports you start earlier and more naturally. Many get stuck doing tennis lessons for three-four years and then drop out.

(Coach 4)

On the question of the number of competition juniors in the clubs, the coaches said it can be difficult to define what a competition junior is and how active these competition juniors really are:

Out of our competition juniors, it is probably only half of them that actually compete regularly. If you have played a match then you are suddenly a competition junior. We have 70–80 competition juniors I think or how many are there really?

(Coach 12)

While the coaches commented on the reduced amount of competition juniors, it was also emphasized that the clubs, both in terms of adults and juniors, are bigger than ever. In some of the clubs, the number of interclub teams for adults was also larger than ever, where in some clubs there were 50–60 competition playing adults and 10–15 interclub teams for adults. This was perceived by the coaches as something positive for the clubs and Swedish tennis, but at the same time it was seen as an obstacle to developing more active competition juniors in the long run. Coach 6, for example, pointed to the shift of focus groups in the clubs:

Before the juniors were in focus, now a lot of focus is instead placed on the adults. They are often very demanding and take up a lot of time and energy from us coaches.

(Coach 6)

Many of the coaches claimed it was the “economy that rules” (Coach 2) and the clubs were sometimes compared with a “factory”:

In the past we had 400 juniors in our club, where 90 percent of them only played once a week and only for 45 min. It was like a factory, so we scaled back, expanded our facilities and hired more coaches, but now it is completely full again so we have to come up with magic again.

(Coach 3)

### 3.3. Everyday Practices for Athlete Development

In the coaches’ stories of how they worked in their everyday practice to create an athlete development environment within the framework of the club’s activities, they said that:

The big focus for me is to work from below... work meticulously already in tennis school... we can’t have an 18-year-old who serves with the wrong grip... we work a lot with joint planning at younger ages, then when they are older more individually...

(Coach 9)

We work clearly with six different levels… for example blue, red, orange and green levels… different checkpoints along the way that we have for all children to see where they are in their development for example forehand, footwork, follow-up…. this to have the best opportunities to develop into a good competition player…

(Coach 11)

Some of the coaches also mentioned how they work a great deal on making sure that their athletes have the opportunities to keep developing their game as they get older:

I work a lot with the collaboration between club and high school to free up time for tennis for the high school students who have high ambitions... I also work with sponsorship to be able to provide them with money to continue to travel and participate in competitions...

(Coach 6)

The coaches also described how the focus on different internal matches is an important part of athlete development:

We have from the beginning a system that is based on playing matches, so that the players get used to playing matches but in a nicer format, such as the “Friday game” and now also Saturday matches which are exchanges with other clubs nearby … The whole thing is about playing a lot of matches so you get used to it.

(Coach 7)

For some of the coaches, the work with creating an athlete development environment was very much about organizing and watching interclub games and competitions:

My work with this is a lot on the weekends, participate at various interclub games and tournaments, where I can follow the juniors’ matches… I often work six days a week or more…. I also follow interclub games and competitions at home.

(Coach 9)

Coach 6 continued to talk about the importance of the athletes learning how to take care of themselves at competitions and camps:

They grow tremendously on these competition trips... the juniors need to feel that it is their sport... too much nagging from the parents sometimes... we do not bring parents on any competitions and camps... these competitions and trips important for the players who have participated…but it also raises the average level for everyone else in the club...so has sort of a flow-on effect.

(Coach 6)

In the coaches’ statements it was also noticeable that it differed to what extent the coaches felt they had the opportunity to be with their athletes at competitions and interclub games:

I travel a bit with the 12–13-year old’s… want to see them playing proper matches… when you see a good even tennis match there is a lot that you can learn and bring home… but I cannot be gone 4–5 days from the club very often, it just doesn’t work.

(Coach 11)

Some of the coaches accounted for how their working days and tasks were largely filled by various “time thieves” (Coach 1). Many of the coaches argued that, in their everyday work, there were a number of these so-called “time thieves” and “administrative” tasks (Coach 5), meaning that time for athlete development and free time to devote themselves to the most active and aspiring athletes did not exist.

Time thieves do not allow time for player development... player development is done in evenings and weekends, one must set aside time to hide away and work on this otherwise it just does not happen.

(Coach 1)

There is not enough time for all the administrative tasks... emails you have to answer… but not fully develop the younger players…. difficult with individual support and development… difficult to plan ahead and have a plan for each player... wish you had more time for it... difficult to follow up training diaries and match reports…

(Coach 5)

### 3.4. Goals for Athlete Development

When the discussions in the focus groups moved towards the clubs’ and coaches’ specific goals for athlete development, some of the coaches talked about how their clubs had some form of goals regarding the development of elite athletes, such as:

We have an explicit goal of enhancing competition players.

(Coach 2)

Goals…. Well we are a club for everyone, everyone is allowed to participate... we want to be able to produce good juniors nationally... we want teams in the highest leagues, but what are the highest leagues?

(Coach 4)

In most cases, however, these goals often seemed to be rather general and not clearly stated and/or written down. In some cases, there were no stated goals regarding the clubs’ activities:

Goals, well to produce as good tennis players as we can produce... but no requirement for having, for example, two international players... we work with the big masses hoping some of them will turn out to be good...

(Coach 7)

No direct goals... you work up to 18 years old and then it ends... no short-term or long-term goals, there are no goals in the club other than what is stated on the website.

(Coach 10)

Goals for developing elite athletes were also seen by the boards of some clubs as in competition with the economy:

A competition situation arises … Should we prioritize economy or competition? Then economy always wins.

(Coach 13)

A recurring theme in the coaches’ statements was also the clubs’ boards often lacking insight into and an understanding of what it means and what is required to develop elite athletes.

Certainly, we have an explicit goal of producing competition players, but dilemma with the board’s interest, do they have knowledge of what it takes to make this journey?

(Coach 2)

The rotation of board members, ignorance, and unwillingness in the board regarding the development of elite athletes as they “cost too much” (Coach 7) is another recurring theme in the coaches’ statements. As one of the coaches explained:

For the board, the economic thing is by far the most important…. very little talk about the competition bit…. when I write the head coach report, I try to highlight the competition juniors a little extra because they did well but it is noticed very little… there is no communication or understanding what is required to be a competition player… we have different experiences of competitive tennis... I have a good understanding of what is required... I have traveled a lot... But have difficulty communicating to those who do not understand or have that interest.

(Coach 12)

When the discussions continued about the goals and focuses of the clubs’ activities and the development of elite athletes, the coaches came up with broader issues, such as the development of the competition aspect. The coaches said the number of juniors is greater than ever, but that, at the same time, this mainly involves a lot of “juice and cookie tennis school” (Coach 4) and that many end up dropping out early at what Coach 9 calls the “11-year hump”. This is what some of the coaches had to say about this:

The number of juniors is greater than ever... but what do we do with them?

(Coach 6)

Tennis is about playing matches... last year we had a club champs for 9–12-year old’s, most of them did not even know the rules, we had to have an umpire for it to work...

(Coach 7)

We have no direct plan... you work up to 18 years then it ends... big numbers and big business between 6–11 years then a sudden hump... most of them drop out.

(Coach 9)

### 3.5. Collaboration and Communication

A further central theme in the coaches’ perceptions regarding their work with creating an athlete development environment in the long term was the need for increased collaboration and communication. The coaches reflected on various initiatives for collaboration with other clubs, coaches, and the regional tennis association but, while some collaborations existed, the coaches agreed that more, extended, and “prestigeless” (Coach 6) collaborations were required:

We can get much better in Swedish tennis at cooperation... work over club boundaries more... must drop this whole thing that this is my club... we have to be more prestigeless…must be more realistic what we can do ourselves for the greater good of the juniors...

(Coach 6)

Some of the coaches further mentioned the need for a regional center for players and coaches. These coaches believed this was necessary to create several regional and national training facilities for players and coaches to meet, train, and collaborate, which then could provide a “ripple effect” (Coach 7) in the clubs.

The fact that we do not have a regional centre is not good... we need somewhere where coaches and players can train together and communicate with each other.

(Coach 1)

We desperately need regional and national centres... where coaches and players can meet up say once a month to train and collaborate.

(Coach 8)

During the conversation about collaboration and communication, the coaches also addressed the need for clearer common guidelines and models for the training and development of athletes at all levels instead of everyone “running their own race”:

As it looks now, each club sits in their own office and re-invents the wheel, why does not the association send out all the expertise that exists…centrally providing content and material what to work with at different ages, as they do for example in France.... if you do not work with this, you lose your funding…but no everyone still sits in their own office and runs their own race... makes us all lose a lot of time and resources that could instead be put into time player development.

(Coach 7)

The coaches also emphasized that excellence, experience, and knowledge already existed in Swedish tennis, but needed to be highlighted, clarified, and made available to all clubs, regardless of size and geographical location:

There are many highly talented and experienced coaches in Sweden who can contribute knowledge and skills…would be good with guidelines…not exactly followed but as a common starting point.

(Coach 1)

Other coaches pointed to the need to utilize knowledge from other countries to a greater extent. Examples were given of coaches from Serbia and other countries who had already visited on a number of occasions and had been training with clubs and coaches involved in the study. The coaches claimed, however, that this type of exchange and knowledge should be spread and distributed better throughout the country. Here, they argued that both the regional and the national association had an important function to fill, as Coach 12 said:

Now we all develop our own checkpoints, it would be better for the national association to develop it... Hire a good coach who, for example, develops an under 10 year olds program that then goes out to other clubs... One region already has its own tennis technique handbook... would be better if we all had a common model... Then of course we can develop it ourselves in our own context... But optimal if it came from the top where the most knowledgeable people are.

(Coach 12)

## 4. Discussion

The aim of the present study was to investigate Swedish tennis coaches’ everyday practices for creating athlete development environments. More specifically, the study aimed to examine what environmental factors promote or hinder athlete development in Swedish tennis clubs, according to professional coaches. In this section, the study’s results will be analyzed and discussed with reference to Henriksen’s ATDE [35] and Fahlström et al.’s UPF [36] models and previous research.

### 4.1. Management and Coaches

Based on the coaches’ responses above, it is clear that the creating of an athlete development environment is largely controlled by the management’s work and the coaches’ opportunities to allocate time and resources. These results particularly highlight how the coaches’ everyday practices are strongly influenced by the management [36], which in this study equates to the tennis clubs’ boards. The board has a great influence on both the focus of the club activities and the coaches’ work situations, which, in turn, affect the potential of the athlete development environment. It is common in the coaches’ statements that an increased focus on the breadth of activities, along with a strong economic focus, is experienced by the coaches to create conditions that often tone down the ambition of encouraging and developing elite athletes. According to many of the coaches in the study, an understanding and knowledge of what is needed to develop elite athletes is also missing. It is perceived as difficult to communicate and discuss what it takes to become a skilled tennis player. Knowledge and awareness of the ability of the environment to develop athletes are highlighted by Henriksen [35] as vital to athlete development work. In addition, the lack of clear job descriptions for the coaches, together with conflicts of interest with the club’s boards, leads to clubs often missing a clear and coherent organizational culture, the importance of which is emphasized by Henriksen [35]. Here, it is important to note that coaches often have their own goals in this regard, but a common and coherent picture of it throughout the club is limited.

The recruitment of coaches and the creation of conditions within which the coaches operate is of crucial importance for the athlete development environment [35,36]. According to what the participating coaches in this study experience, it is usually the best, most capable, qualified, and experienced coaches who have a wide range of responsibilities that end up doing a lot of administrative work, rather than spending time on the court and in athlete development. In addition, managing the large amount of part-time coaches, along with other extra tasks (e.g., being caretakers, competition organizers, sponsor recruiters) seems to have a negative impact on the coaches’ opportunities to work on athlete development. Some of the coaches also indicate how these administrative and extra tasks lead to an unhealthy work situation, which might endanger their long-term health and well-being. In reiterating the need for a holistic approach to athlete development environments [5,35], this calls for further examinations of how coaches’ health and well-being can be seen as another environmental factor that can potentially promote or hinder athlete development.

Focusing on the long-term perspective regarding athlete development is something that also seems to be lacking, especially when it comes to the goals and visions of the club. Other focuses in the club, such as the economy and breadth of activities, along with lack of time, lead to a limited athlete development environment. As such, this study highlights how two of the important environmental factors identified by Martindale et al. [5], “consensus in communication and support” as well as “long-term goals and methods”, seem to hinder athlete development in the participating coaches’ tennis clubs.

### 4.2. The Immediate Surrounding and Developing Environment

Within the other levels of the athlete development environment—the “immediate surrounding”, and the “developing environment”, as shown in Figure 2—the results of this study point to a number of different environmental factors related to communication and collaboration that seem to either promote or hinder athlete development. It is precisely the factor “related clubs/teams”, within the immediate surrounding environment level, that the conversations with the coaches in the interviews revolve around. In particular, it is the communication and cooperation with other coaches, clubs, regions, academies, regional centers, and associations that is raised. One of the things that the coaches emphasized particularly during the focus groups is the desire for more cooperation, communication, and “prestigelessness” between clubs, coaches, players, parents, and regional and national associations. Indeed, both Henriksen’s [35] and Fahlström et al.’s [36] models emphasize the importance of an athlete development environment being based on collaboration, openness, and knowledge exchange.

Another factor linked to the immediate surrounding development environment [36] is the school. Here, the increased number of dropouts from tennis before or in connection with high school is highlighted. This can be considered as particularly problematic, since studies in recent years have shown it is just below the high school age that future national representatives and other successful athletes specialize in their sport and start increased effort, which then leads to elite performances [15]. In the coaches’ statements, recurring problems are mentioned with both trying to keep the athletes in tennis throughout high school and to provide those continuing with good opportunities for training and competition. In this area, therefore, more work and knowledge is required to create an environment within tennis clubs that can be supportive in several ways, instead of acting as an exclusion factor when the athletes make the choice to continue with tennis and develop further or to drop out to pursue other things, such as work and studies [7,21]. Part of this could be about giving the athletes more support and resources for their studies, combined with the development of a tennis career to facilitate the athletes’ transition from junior to senior tennis, as this step is often perceived as difficult and stressful by young athletes [34]. Another important part of this could involve adopting a person-centered approach, where the clubs and coaches together with every athlete work actively with short- and long-term goal setting, while taking into account the athlete’s overall health and well-being [38].

The desire for clearer common guidelines and models, as well as the dissemination of knowledge from the national association regarding “best practice” is another recurring theme in the coaches’ responses. These common guidelines for best practice should include what the training program involves at different ages/stages, where the importance of both taking care of the knowledge that already exists internally within the country/national association and the obtaining of external “expert” knowledge from other countries is emphasized. The coaches mentioning of “experts”, as shown in Figure 2, further highlights this role as another vital factor in the overall athlete development environment [36]. As a continuation of the focus on cooperation and communication regarding, for example, common guidelines and the utilization of internal/external expert knowledge, the need for extended national and regional training facilities is highlighted. Here, athletes and coaches can come in different periods to train with and learn from the expertise that national and regional associations can offer. When it comes to this cooperation between coaches, clubs, regions, and the national association, it may be good to have Brown’s [8] study in mind, showing that developing elite athletes require different types of coaches during different periods. This can then vary from “local” coaches who can offer a lot of time and encouragement—coaches who can help the athletes improve their technical and tactical skills—to licensed coaches who themselves have played at an international level. A clear structure and plan for cooperation between local clubs/coaches and regional, as well as national, coaches could offer this form of athlete development environment. This would then, like Alfermann and Stambulova [34] argue, constitute the overall athlete development environment in which the athletes are prepared for and developed into elite athletes. These results demonstrate the importance of a well-established and systematically implemented sport policy as an environmental factor in creating successful athlete development environments [39].

Finally, it should be noted that the participating coaches in this study demonstrate knowledge, commitment, and interest in their own continuous professional development [26,27]. The importance of this is emphasized in the studies by both Gilbert and Trudel [28] and Denison et al. [29], who believe it is important that coaches have a positive attitude towards lifelong learning in a sport that is constantly evolving, and that they always have a reflective approach towards their own work. However, the coaches’ responses signal that the conditions and opportunities for continuous professional development in the form of education and participation at coaches’ conferences/workshops seem limited. A lack of resources and time within their coaching roles seems to constitute the biggest obstacles. According to Mallet et al. [30], a constant development is a prerequisite if coaches are to be high-performing and thus able to develop high-performing athletes. The limited opportunities for professional development have thus been identified in this study as another environmental factor that hinders the potential of the athlete development environment and that could be adapted by sport policies and management work.

## 5. Conclusions

This study has investigated Swedish tennis coaches’ everyday practices for creating athlete development environments and how different environmental factors promote or hinder athlete development. The results of this study confirm the central role that the “management” level plays in creating the overall athlete development environment, as proposed in Fahlström et al.’s [36] UPF model. The management (tennis club board) has a great impact on both the focus of club activities and the coaches’ work situations, which in turn affect the coaches’ everyday practices and ultimately the potential of the athlete development environment to develop elite athletes. The present study shows that there are shortcomings in both clear job descriptions and goals regarding the development of elite athletes. This lack of clarity about job requirements and goals, together with a focus on a club management (board) that often prioritizes economy and high participation levels, lead to the coaches having limited time and resources to focus on athlete development. The results also draw attention to concerns about these professional coaches’ health, with increasing demands in their roles to manage administrative tasks in addition to the coaching and time spent on the court with their athletes. In terms of the socio-economic, cultural, and sporting conditions that also govern the athlete development environment, according to Fahlström et al.’s [36] UPF model, the coaches in the present study emphasize the importance of increased collaboration and communication between clubs, coaches, regions, and the national association to create common and clearer guidelines for long-term athlete development. Future studies could engage in longitudinal and ethnographic work with tennis clubs of varying sizes and geographical locations involving different stakeholders (e.g., coaches, management, parents, players) in order to further explore the environmental factors that promote or hinder athlete development.

## Figures and Tables

**Table 1:** The number of full-time tennis coaches, members, juniors, and competition juniors in participating coaches’ clubs.

Coach/Club	1	2	3	4	5	6	7	8	9	10	11	12	13
Full-time coaches	2	3	6	5	3	5	8	2	2	1	2	2	2
Members	600	860	1100	1100	600	1000	1800	3000	800	300	1300	7000	400
Juniors	300	300	400	450	190	280	300	600	300	180	500	300	140
Competition juniors	50	30	60	20	20	15	50	70	30	20	80	30	10
